# Noble Metal Nanostructured Materials for Chemical and Biosensing Systems

**DOI:** 10.3390/nano10020209

**Published:** 2020-01-25

**Authors:** Mingfei Pan, Jingying Yang, Kaixin Liu, Zongjia Yin, Tianyu Ma, Shengmiao Liu, Longhua Xu, Shuo Wang

**Affiliations:** 1 State Key Laboratory of Food Nutrition and Safety, Tianjin University of Science & Technology, Tianjin 300457, China; panmf2012@tust.edu.cn (M.P.); yangjy0823@126.com (J.Y.); lkx13642168374@163.com (K.L.); yinzongjiasiss@126.com (Z.Y.); maty1128@126.com (T.M.); lsm20000711@outlook.com (S.L.); 2 Key Laboratory of Food Nutrition and Safety, Ministry of Education of China, Tianjin University of Science and Technology, Tianjin 300457, China; 3 School of Food Science and Engineering, Shandong Agricultural University, Shandong 271018, China; longhuaxu@sdau.edu.cn

**Keywords:** noble metal nanostructured materials, chemical sensing, biosensing, review

## Abstract

Nanomaterials with unique physical and chemical properties have attracted extensive attention of scientific research and will play an increasingly important role in the future development of science and technology. With the gradual deepening of research, noble metal nanomaterials have been applied in the fields of new energy materials, photoelectric information storage, and nano-enhanced catalysis due to their unique optical, electrical and catalytic properties. Nanostructured materials formed by noble metal elements (Au, Ag, etc.) exhibit remarkable photoelectric properties, good stability and low biotoxicity, which received extensive attention in chemical and biological sensing field and achieved significant research progress. In this paper, the research on the synthesis, modification and sensing application of the existing noble metal nanomaterials is reviewed in detail, which provides a theoretical guidance for further research on the functional properties of such nanostructured materials and their applications of other nanofields.

## 1. Introduction

Nanomaterials, also known as nanostructured materials, generally refer to nanocomposites assembled in a three-dimensional structure with at least one dimension in the nanometer size range (0.1–100 nm) or nanostructured materials as basic constituent units [1,2]. This kind of materials usually has large specific surface area, controlled surface activity, special quantum size effect, surface/interface effect, macroscopic quantum tunneling effect, photoelectric effect, catalytic effect and volume effect [3,4]. These unique properties have given nanostructured materials excellent optical, electrical, magnetic and other characteristics, making these materials widely concerned in various research fields [5,6,7].

Noble metal nanomaterials are an important part of nanostructured materials, which have shown a wide application space in the fields of new energy, photoelectric information storage and functional catalysis [8,9,10]. This kind of materials combined the special physical and chemical properties of noble metals with nanomaterials to exhibit superior performance, expanding the application range of nanomaterials. It mainly covers several aspects as follows: (1) as a highly efficient catalytic material [11,12]; (2) as a highly conductive material in the sensing field [13]; (3) with large specific surface area for energy storage [14,15]; (4) introduced into a multi-metal composite material [16,17]. To date, a large number of noble metal nanomaterials with different morphologies (balls, rods, flowers, sheets, cages, etc.) have been developed to meet the requirements of different research [18,19]. Additionally, metal-based nanomaterials are easy to prepare and modify on the surface and obtain controllable properties. The structure, morphology and size of metal nanomaterials can be adjusted and optimized to obtain the special photoelectrochemical properties, called special size dependence, making them very promising in various sensing fields [20,21,22].

In this paper, the synthetic methods and functional properties of various nano-structured materials of noble metals are reviewed in detail. The applications of typical noble metal nanomaterials with different properties for chemical and biosensing systems are also introduced and discussed. This paper has great significance for the study of the functional properties of different metal nanomaterials and expand their applications in various research fields, especially in sensing fields.

## 2. Synthesis of Noble Metal Nanomaterials with Different Morphologies

### 2.1. Nanoparticles (NPs)

Au-based nanomaterials, one of the earliest nanostructured materials, are one of the research hotspots in the field of nanoanalytical science, which have been extensively studied [23,24]. Various Au-based nanomaterials with different physicochemical properties including spheres, rods, nanoclusters and wires have been successfully prepared, leading to the change in signal form or strength in analytical system. This is the basis of sensing analysis and bioimaging research [25,26]. After nearly three decades of research and exploration, various methods to prepare AuNPs have been developed and can be roughly divided into physical and chemical methods. AuNPs prepared by physical methods (e.g., grinding and evaporation) are usually large in size, and the requirements for production equipment are relatively harsh. This is not conducive to their application in sensing systems. The process of chemical preparation of AuNPs is relatively simple and low-cost. Generally, a suitable reducing agent (e.g., sodium borohydride, sodium citrate, ascorbic acid, etc.) is applied to reduce the Au ions or Au-containing compounds in the solutions. By controlling the amounts of reducing agent, protecting agent and solvent, AuNPs of different size and morphology were prepared [27]. AuNPs are usually composed of Au atom as a core and a double ion layer surrounding the outer layer. The double ion layer includes the inner layer of AuCl_2_^−^ and the outer layer of H^+^ for maintaining the steady state of AuNPs in solutions. Sodium citrate is the first substance in the AuNPs synthesis, which functions as both a reducing agent and a stabilizer. However, the particle size of the synthesized AuNPs is mostly larger than 10 nm, and the dispersibility is relatively poor [28,29]. In 1990s, Brust and Schiffrin have combined the self-assemble process with nano technique to prepare monodisperse AuNPs, which were very stable from aqueous phase to organic phase using thiol self-assembled membranes to reduce HAuCl_4_, named phase transfer method. Due to the strong passivation of thiol substances, the obtained AuNPs have relatively small particle size and good stability (no aggregation and decomposition), though the particle size distribution range is relatively wide [30,31]. Furthermore, using a thiol compound such as 5-phenylthiocarbazole or cysteine as stabilizer and a large molecular polymer such as polyhexamethylene glycol, silk fibroin or polyaniline as reducing agent, the prepared AuNPs were with a uniform particle size and suitable for the construction of sensing systems with high performance [32,33].

**Figure 1:**
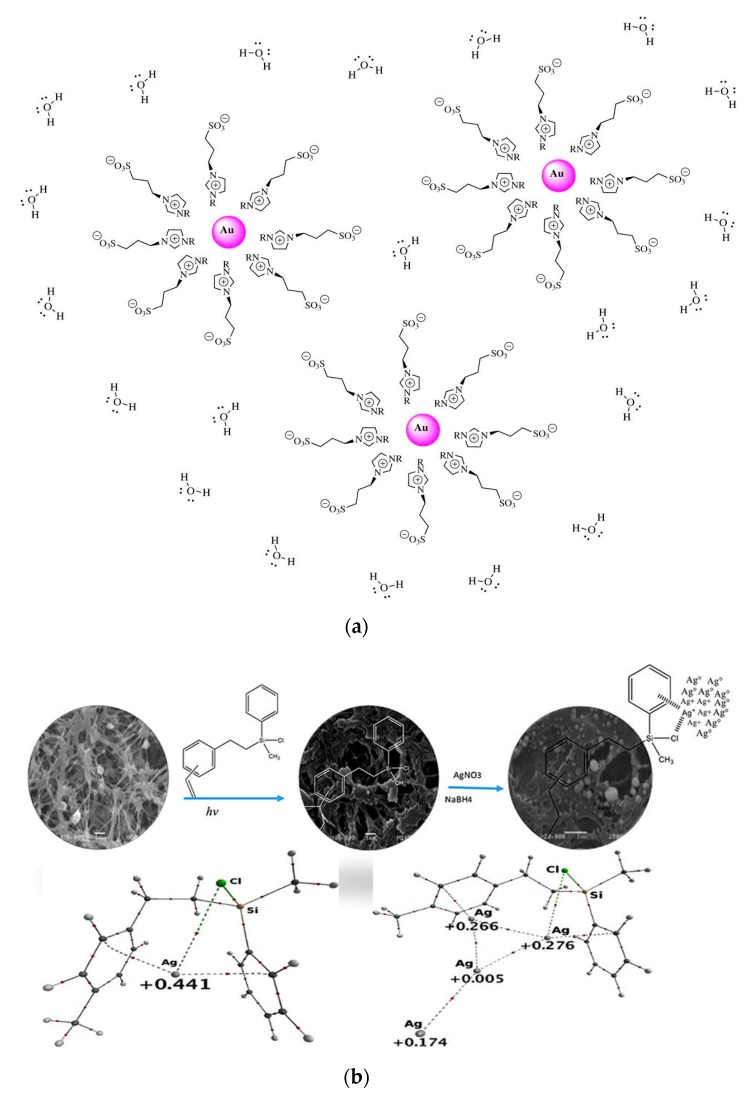
(**a**) Scheme of AuNPs stabilized with imidazolium salts in water. Reproduced with permission from [37]. Copyright Royal Society open science, 2017.; (**b**) Synthesis of PP-ECMPS and used as a template for the synthesis of AgNPs. Reproduced with permission from [40]. Copyright Chemical Physics Letters, 2018.

Reverse micelle or microemulsion method refers to a heterogeneous liquid phase synthesis method for forming micelles or reverse micelle droplets between a surfactant and a solution for restraining the size or morphology of NPs [34]. The surfactant can disperse the NPs well, making them less prone to agglomeration and precipitation. This method is easy to control and operate, and overcome the shortcoming of wide particle size distribution of NPs product. However, because large amount of surfactant is used, the purity and yield need to be improved [35,36]. Monti et al. reported the synthesis of AuNPs in a confinement environment created by reverse micelles using sulphonated imidazolium salts [37] (Figure 1a). In homogeneous media, AuNPs interacted with the imidazole ring, while the sulphonate groups were far away from AuNPs surface. The better stability of the obtained AuNPs indicated that sulfonated imidazole salt was a very effective stabilizer.

Templating method generally employs a matrix with micropores or mesopores (e.g., silica, high molecular polymer) as a template, and a chemical reduction reaction in the micropores to form a nanomaterial. This method can obtain metal NPs with narrow particle size distribution [38,39]. Mendieta-Jimenez et al. have employed [2-(vinylphenyl)ethyl] chloromethylphenylsilane (ECMPS) to modify the polypropylene (PP) membrane to obtain growth sites. The obtained PP-ECMPS were applied as the template for AgNPs growth by chemically reducing AgNO_3_ by NaBH_4_ [40] (Figure 1b). PtNPs were synthesized through a double-template electrochemical deposition and used as electrochemical interface for electrocatalytic reduction of hydrogen peroxide (H_2_O_2_) and electrocatalytic oxidation of N_2_H_4_, displaying a fast response (<4 s) and high sensitivity (110 µA mM^−1^ cm^−1^) [41].

### 2.2. Nanorods (NRs)

**Figure 2:**
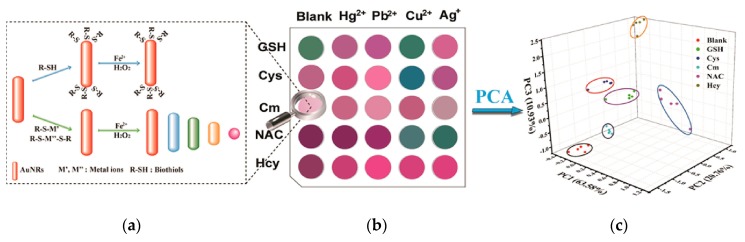
Label-free AuNRs sensor array for colorimetric detection of biothiols. Reproduced with permission from [47]. (**a**) Synthesis of various AuNRs; (**b**) Fingerprint-like colorimetric array for metal ions; (**c**) Principal component analysis. Copyright Talanta, 2019.

As one specific form of NPs, NRs generally with a capsule shape have more significant properties. Taking as an example, AuNRs have the absorptions of transverse about 520 nm and longitudinal surface plasmon resonance (SPR) [42]. Thus, by changing the radial ratio of NRs, the SPR adsorption can be controlled red-shift from the visible region to the near-infrared region (NIR), achieving optical transmission in the biological samples [43,44]. Additionally, the localized SPR (LSPR) of AuNRs is very sensitive to the dielectric constant of the surrounding environment, which have remarkable advantage in the construction of non-labeled sensing system [45,46]. Yuan et al. employed label-free AuNRs composed of metal ions (Hg^2+^, Pb^2+^, Cu^2+^, Ag^+^) as the colorimetric probe (output signal) to establish a nanosensing array for biothiols. Based on the different binding affinity of biothiols and metal ions, AuNRs exhibited a unique pattern to form a fingerprint-like colorimetric array. By combining with principal component analysis (PCA), this array was able to discriminate five biothiols by the naked eyes [47] (Figure 2).

**Figure 3:**
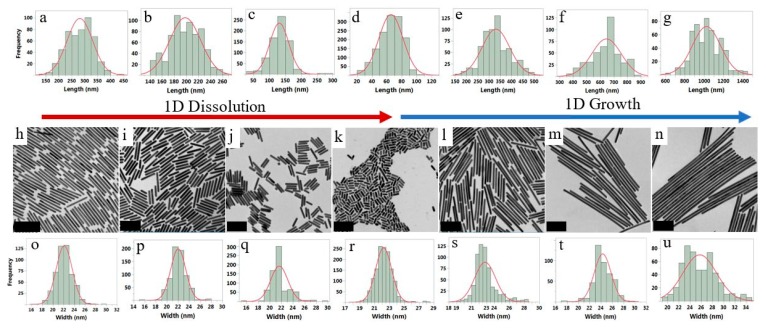
The length and width distribution of AuNRs during the dissolution and growth process and the corresponding transmission electron microscopy (TEM) images. Length distribution (**a**–**g**), width distribution (**o**–**u**) and corresponding TEM images (**h**–**n**) during the growth and dissolution processes. Reproduced with permission from [59]. Copyright ACS Nano, 2019.

At present, many synthetic methods have been reported to prepare NRs with good dispersibility and uniform particles. Seed growth method is one of the most widely used and most thoroughly studied methods [48]. The basic process is adding a certain concentration of seed crystals to the growth liquid, and under the action of the surfactant, orienting the seed particles to grow into NRs. The yield and radial ratio of NRs can be controlled by adjusting the ionic strength (pH) of the solution [49,50]. Zhang and his co-workers synthesized a tunable-aspect ratio AuNRs using a modified seed-mediated synthesis method for near-infrared photoacoustic imaging [51]. Wang et al. applied the seed-mediated growth to achieve a high sensitivity due to the low energy barrier in the target-induced formation of bimetallic NRs with core-shell structure. In alkaline solution, Ag^+^ can be reduced to Ag atoms and deposited on the surface of AuNRs to generate Au@Ag core-shell NRs, accompanied by blue shift of the LSPR of AuNRs from near-infrared region to shorter wavelengths [52]. The templating method can also synthesized NRs with better dispersibility [53,54,55]. In the synthesis of Au/Ag NRs, the use of soft template received more attention because it does not require subsequent removal of the template. It is worth mentioning that during the whole process of preparing NRs, the parameters including the pH of the solution, the type and amount of the surfactant and coating agent have a significant influence on the radial ratio of NRs, which need to be very tightly controlled [56]. Cetyltrimethylammonium bromide (CTAB) is the most commonly used surfactant and can form rod-shaped micelles in solution, which can effectively control the directional growth of anisotropic nanomaterials and promote the formation of NRs. In this mode, the CTAB acts as both a template and coating agent, facilitating mass production of NRs and nanowires [57,58]. Khanal et al. developed a reversible chemical process for tip selective one-dimensional growth and dissolution of AuNRs, which can be continued with additional supply of Au(I)/CTAB/ascorbic acid solution to produce extremely long Au nanowires [59] (Figure 3).

Electrochemical method is a process of applying a certain current or voltage to a noble metal electrolyte system to cause an electrochemical reaction (anodization, electrolysis, deposition) to obtain a noble metal nanomaterial. Among them, electrochemical anodic oxidation can effectively regulate the size and morphology of nano-products by controlling the experimental conditions such as electrode voltage, electrolyte composition, ionic strength and time [60]. Adding a coordination stabilizer into the electrolyte solution can be effectively prevented from agglomeration of particles and control the nanostructure. Electrochemical ultrasonic method refers to the fact that metal can be quickly dispersed into the entire electrolyte under ultrasonic energization, and prevent the nanomaterials from further growing, the obtained nanomaterial has a relatively small particle diameter [61,62]. Photo reduction method does not require heating at high temperature, and can control the size of nanomaterial by adjusting the power of the light source. Bandita et al. reported one sun-light driven rapid green synthesis of stable aqueous dispersions of AgNPs and AgNRs at room temperature [63]. Through the photoreduction of Ag^+^ with Piper nigrum extract, the Ag nanomaterials were formed within 3 min of sun light irradiation. Majid’s group present the formation of AuNRs on novel Au-poly(methyl methacrylate) nanocomposite substrates, which is formed by ultraviolet (UV) photoreduction. [64].

### 2.3. Nanocage

**Figure 4:**
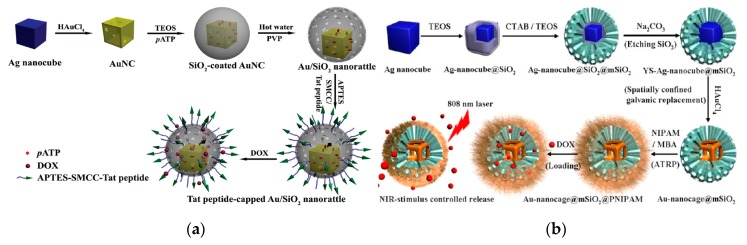
(**a**) Schematic diagram of the synthesis of the double-walled Au/SiO_2_ nanorattles. Reproduced with permission from [71]. Copyright small, 2015; (**b**) Fabrication of the Au nanocage@mesoporous SiO_2_ core-shell structure for NIR-stimulus controlled drug release. Reproduced with permission from [72]. Copyright Chemistry of Materials, 2013.

Nanocage refers to a three-dimensional nanomaterial with a hollow interior and a porous outer shell, which can effectively perform intramolecular assembly and external modification. The current research on metal nanocage is mainly focused on Au nanocage. This is mainly because Au nanocage has good light scattering, light absorption and unique LSPR peak in the NIR, which has unparalleled advantages in optical imaging [65,66]. At the same time, Au nanocage has photo-responsive properties, which is easy to respond sensitively to external stimuli (temperature, pH, etc) [67]. Au nanocage with low toxicity and good biocompatibility has the outer surface easily modified by biomolecules and organic macromolecules. The surface area for light-absorbing is also much larger than that of the traditional dye molecules (about 10^5^ times) [68]. Based on these advantages, Au nanocage has been widely used in the fields of drug controlled release systems [69], biosensing [70] and enzyme immobilization. Wang’s group has fabricated the double-walled Au nanocage/SiO_2_ nanorattle by combining two “hollow-excavated strategies”—galvanic replacement with “surface-protected etching” [71] (Figure 4a). The Au nanocage acts as not only a sensitive surface enhanced raman spectroscopy (SERS) substrate to track the internalization process of the nanorattles by human MCF-7 breast cancer cells, but also an efficient photothermal transducer for localized hyperthermia cancer therapy due to the strong near-infrared absorption. Yang et al. has fabricated a novel multifunctional NIR-stimulus controlled drug release system with Au nanocage as photothermal cores, mesoporous SiO_2_ shells as supporters to increase the anticancer drug loading and poly (*N*-isopropylacrylamide) (PNIPAM) as NIR-stimuli gatekeepers to controll drug release [72] (Figure 4b).

### 2.4. Nanoclusters (NCs)

Compared with the other Au nanomaterials, Au nanoclusters (AuNCs) at smaller particle size can generate fluorescence under a certain wavelength of light excitation, and have strong anti-photobleaching and good water solubility. The wavelength of emitted light can vary with cluster size. These superior properties make AuNCs as fluorescent probe for sensing and imaging that greatly enhance the selectivity and sensitivity and improve the imaging capability of biomarker [73,74]. The physical method for preparing AuNCs is dispersing large pieces of Au particles into nano-sized metal particles, and the obtained AuNCs have a wide particle size range and poor light stability. The chemical synthesis method using specific molecules as template can effectively control the size of AuNCs. Now, the proteins, peptide, poly(amidoamine) dendrimer (PAMAM), and DNA were all reported to be used as template [75,76,77,78].

**Figure 5:**
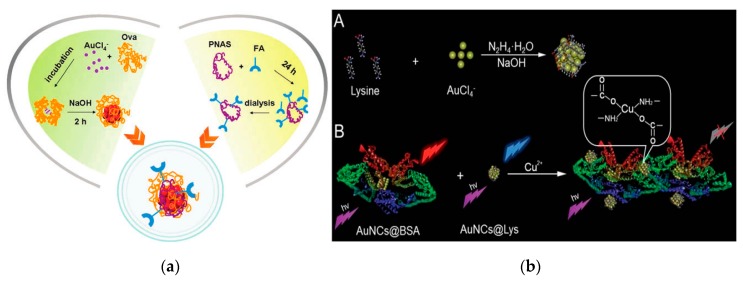
The proteins used as template molecule for metal NCs synthesis. (**a**) Schematic of the formation of AuNCs using ovalbumin as template. Reproduced with permission from [81]. Copyright Chemical Communications, 2013.; (**b**) Scheme of the synthesis of lysine-stabilized AuNCs and the strategy for Cu^2+^ detection. Reproduced with permission from [84]. Copyright Journal of Materials Chemistry C, 2013.

Since most proteins and peptides have binding sites that can chelate and reduce with metal ions, AuNCs can form and stably exist by adhering to these binding sites. In 2009, Ying et al. reported the use of bovine serum albumin (BSA) as template to synthesize AuNCs (only 25 Au atoms) that can emit intense red fluorescence [79]. This study not only provides a green and simple synthesis method for AuNCs, but provides a new idea for other proteins as template molecules to synthesize other metal NCs. Nowadays, the protein of horseradish peroxidase (HRP) [80], ovalbumin [81] (Figure 5a), lysozyme [82] and trypsin [83] have been used as a template for metal NCs synthesis. Yang’s research group synthesized highly fluorescent lysine-stabilized AuNCs by a simple and environmental friendly approach for sensitive and selective detection of Cu^2+^ [84] (Figure 5b).

**Figure 6:**
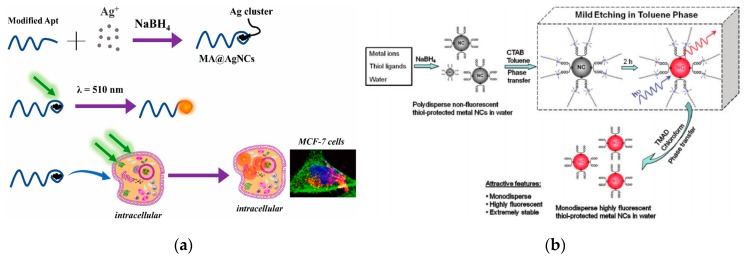
(**a**) Scheme of modified aptamer@AgNCs synthesis process and application on fluorescence cell imaging. Reproduced with permission from [86]. Copyright Talanta, 2019. (**b**) Scheme of the process to generate highly fluorescent metal NCs by reversible phase transfer. Reproduced with permission from [92]. Copyright ACS Nano, 2011.

The DNA long chain contains a specific base heterocyclic ring, and a strong metal-based bond to obtain a high affinity with metal ions [85]. NCs synthesized using DNA as template have good biocompatibility and specific recognition ability, and the synthesis process is simple and controllable. Wang et al. synthesized fluorescent AgNCs using 12 cytosine based sequence and the obtained AgNCs shows descent specific recognition of MCF-7 cell, which have potentially utilized for clinical diagnosis and treatment [86] (Figure 6a).

In addition to biomacromolecules as template, macromolecular polymers containing large amounts of –COOH and –NH_2_ can also be used as template for NCs synthesis. This kind of polymer is demonstrated to be an electrolyte with better hydrophilicity, and the three-dimensional network structure formed by –COOH and –NH_2_ can protect NCs during the synthesis process. The PAMAM dendrimer is a tightly-spaced spherical molecule with a broad cage cavity inside, and has a large amount of reactive functional group (–NH_2_) on the outer surface [87]. The noble metal ions can be concentrated in the internal cavity of PAMAM molecule by electrostatic adsorption or chelation with reactive functional groups on the surface of the polymer. Due to this “cage” effect, the particle size of the generated NCs can be effectively controlled during the reduction process. By adjusting the ratio of PAMAM to Au, AuNCs containing different number of Au atoms can be obtained, whose fluorescence wavelengths can vary between 380 and 868 nm. This optical property is not available in the PAMAM molecule itself, thus providing the evidence for the preparation of AuNCs of different particle sizes by using PAMAM molecule as template [88]. The thiol-containing compound can form a monomolecular self-assembled layer on the surface of Au nanomaterial through Au-S bond. Based on this, the sulfhydryl-containing small molecule compound can also be used as a stabilizer and a protective agent for AuNCs synthesis [89,90,91]. At present, the method by the protection of mercapto compounds is generally hydrothermal. Xun et al. reported a simple and scalable method for the synthesis of highly fluorescent Ag, Au, Pt, and Cu NCs by the combination of phase transfer hydrothermal and etching method using glutathione for protection [92] (Figure 6b).

## 3. Application of Noble Metal Nanomaterials in the Field of Sensing

The sensing detection is one of the emerging analytical technologies in recent years [93,94]. The recognition element for sensing are generally required to have high selectivity and sensitivity, which usually employ chemical or biological active units such as enzymes, antibodies as sensitive elements. The sensing transducer can recognize chemical or physical signal including light, electricity, and heat etc. Noble metal nanomaterials with unique photoelectric properties and good biocompatibility play an important role in chemical and biosensing system [95,96]. Recently, the research on sensing devices of different principles and signals using noble metal nanomaterials have emerged, which provided a new direction for the development of analytical chemistry and sensing technology [97,98].

### 3.1. Optical Sensor

The unique properties of fluorescence and SPR light absorption of metal nanomaterials make them the most commonly used in analysis. As a result, the application of noble metal nanomaterials in optical analysis in present researches is mainly concentrated at two aspects: one is to quantitatively analyze by monitoring the fluorescence of the nano-colloid solution caused by the analyte or the change of the SPR optical signal intensity, and the other is to achieve the qualitative analysis by characterizing the fluorescence or color intensity change of NPs [99,100,101].

#### 3.1.1. Fluorescence Sensing

For fluorescence sensing, fluorescent probes with high intensity and quantum yield can achieve the same luminescence intensity at low concentrations, resulting in higher analytical sensitivity. Therefore, nanomaterials with strong optical stability and high fluorescent quantum yield are necessary for the construction of the fluorescence sensors with high sensitivity [102,103]. Secondly, the size effect of nanomaterials has a significant impact on the sensitivity of sensor. When the nanomaterial has a volume close to the size of the analyte (small molecule or ions), it is more sensitive and easy to interact between them, which was named the “small-size effect”. This small-size effect allows the fluorescent probe to have a faster reaction rate in the sensing system, which is difficult to achieve using other large-size nanomaterials. Additionally, the generation mechanism of fluorescence signal is another crucial parameter for the fluorescent sensitivity, which depends on the reaction affinity between the active recognition site and the analyte. Higher affinity often improves the efficiency of light signal generation and achieves highly sensitive detection. Therefore, designing a highly sensitive and selective optical sensor based on noble metal nanomaterials relies on an efficient method for preparing nanoprobes holding strong luminescence properties, ultra-small size, and ease to be modified by other ligands.

**Figure 7:**
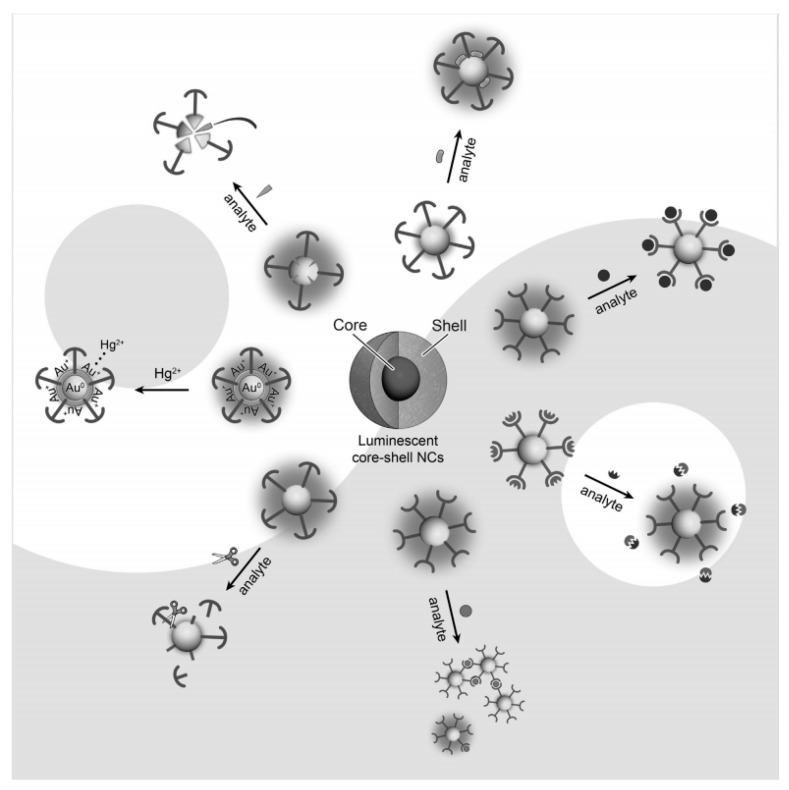
Response mechanisms of current noble metal NCs-based optical sensors. Reproduced with permission from [104]. Copyright Chemistry–An Asian Journal, 2013.

The response mechanism of current noble metal NCs-based optical sensors can be classified into the following two categories [104] (Figure 7): one is using a metal core as recognition site (White part) and the other is using a ligand for recognition (Black). Some specific analytescan etch metal, causing the fluorescence quenching response, which have a significant correspondence with the concentration or amount of the target analyte as the basis for fluorescence quantitative analysis. Heavy metal ions (Hg^2+^, Cu^2+^, Cd^2+^, etc.) in the environment can easily enter and accumulate in the organism, causing irreparable damage to human nerves, reproduction and metabolic systems [105]. The Hg^2+^ has a similar orbital arrangement (4f^14^5d^10^) as Au^+^, resulting in a good quenching effect on the AuNCs fluorescence. Bothra et al. synthesized AuNCs using BSA as soft template and applied to detect Hg^2+^ fluorescence quenching with high sensitivity and selectivity [106]. The limit of detection (LOD) is obtained lower than 10 nmol L^−1^. Wang et al. also prepared bimetallic Au-Ni alloy nanocluster materials using BSA under the conditions of simultaneous presence of chloroauric acid and nickel chloride [107]. Based on the stronger specific bonding ability, Au-Ni alloy NCs can directly detect low concentrations of Hg^2+^ in water, obtaining a wide linear range (0.005–20.0 μmol L^−1^) and a low LOD (1.8 nmol L^−1^).

In addition, fluorescence quenching can also be achieved by depositing compounds or ablating metal cores on the surface of AuNCs. The S^2−^ ions are not only harmful to human body, but also can react with acidic components in the air, which greatly harms the environment. On the basis of the combination of S^2−^ with the Ag^+^ on the surface of AuNCs, a stable Au/AgNCs was synthesized in a high ionic solution using a specific DNA sequence as template [108]. When adding the S^2−^, the interaction with Au/Ag ions or atoms results in a change of DNA template from the hairpin structure to the irregular crimp structure. This DNA conformational change causes core ablation of Au/AgNCs, which leads to fluorescence quenching in a highly sensitive, visual detection system. Biothiols (e.g., cysteine, glutathione, etc.) can specifically interact with Ag on Au/AgNCs surface and cause fluorescence quenching of metal clusters. The developed visual detection sensors using cysteine and glutathionecan detect Hg^2+^ achieving LOD of 5.87 nmol L^−1^ and 1.01 μmol L^−1^, respectively [109]. The stabilizer shell of NCs can effectively prevent the aggregation of NCs core and can be acted upon by active matrix (such as enzyme, peptide), which provides a new analytical idea for NCs with high fluorescence performance. The analyte can be subjected to enzymatic digestion or enzymatic processes to cause the changes in the fluorescence intensity, thereby realizing the fluorescence sensing analysis [110,111]. The HRP molecule was employed as a template to synthesize AuNCs with bifunctional fluorescence. Since the HRP enzyme encapsulated in AuNCs can still catalyze the H_2_O_2_ reaction, resulting in fluorescence quenching of AuNCs to sensitively detect H_2_O_2_ [80]. The stabilizer shell of the NCs canbe modified into recognition sites to obtain efficient fluorescence sensing system. The high fluorescence performance of glutathione-stabilized AuNCs can chelate with Fe^3+^ and quench the NCs fluorescence by efficient energy transfer. Phosphate can recover fluorescence by binding to Fe^3+^, thereby enabling analysis and detection of two targets [112]. Such a method of modifying or changing the structure of NCs stabilizer as recognition signal fully utilizes the specific binding effect of the recognition moiety, greatly improving the selectivity and sensitivity of the fluorescence sensor. The detection mechanisms for quenching fluorescence based on the interaction between the analyte and NCs to cause clustering of the NCs or to disrupt the interaction between the metal core and the ligand have also been reported. However, due to the ultra-small size of noble metal NCs and the contribution of the ligand template, it is generally believed that several response modes exist during the detection process.

#### 3.1.2. UV-Visible Absorption Spectroscopy

**Figure 8:**
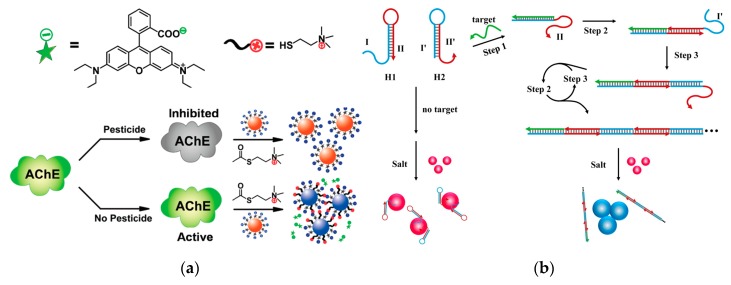
Visual biosensor based on AuNPs. (**a**) Dual-readout assay based on AuNPs for pesticides. Reproduced with permission from [120]. Copyright Analytical Chemistry, 2012. (**b**) Colorimetric detection of DNA by the use of AuNPs and hybridization chain reaction amplification. Reproduced with permission from [121]. Copyright Analytical chemistry, 2013.

Different sizes and shapes impart the unique optical properties of noble metal nanomaterials. Among them, the strong and stable SPR absorption in the UV-visible region is a very important feature. Since the SPR frequency of noble metal NPs can be affected by many factors such as metal type, particle size, morphology and surface charge, its optical properties are not only related to its surface morphology, but alsothe distance between the particles [113,114]. When the distance between the particles is less than its radius, plasma coupling occurs and cause the change in the UV-visible absorption peak. Therefore, it is possible to develop a colorimetric detection using noble metal nanomaterials as probe, and achieve a semi-quantitative detection and quantitative detection by UV-visible absorption [115,116]. Small particle size (1–20 nm) of AuNPs will produce a corresponding color change (wavelength: 520–700 nm).This wavelength change provides a basis for the accurate recognition of some targets, which makes AuNPs have a deep application in sensor detection. The results can be observed directly through simple and rapid colorimetry, having a broad application prospect in the fields of environmental detection, food safety and medical detection [117,118,119,120] (Figure 8a).

The DNA strand has a negatively charged phosphate backbone, and the modification of single stranded DNA (ssDNA) to the surface of the AuNPs can enhance the electrostatic repulsion between the particles, making them stable at high salt concentrations. When the spatial structure of DNA changes, the DNA will move away from AuNPs surface, leading to the agglomeration of AuNPs [122,123]. When the target DNA strand exists, the hairpin DNA originally adsorbed on the surface of AuNPs will be bound to trigger a hybridization and form a longer sequence of double-stranded DNA, which causingthe AuNPs agglomeration appears that the color of the solution changes from red to blue [121] (Figure 8b). For the detection of small molecules, an aptamer (Apt) capable of specifically recognizing the target can be coated on the surface of AuNPs. The ochratoxin can form a *G* quadruplex with the Apt and be away from the AuNPs, causing a color change of the solution to achieve visual detection with LOD of 20 nmol L^−1^ [124]. Some specific metal ions can act as catalyst for nucleases, or can be paired with specific DNA bases (similar to double helix), which was applied in DNA molecular logic gate analysis [125,126,127]. Hg^2+^ can induce thymidine T to form the structure of T–Hg–T, while Ag^+^ can induce the C–Ag–C structure formed by cytosine C. According to this two structures, Zhang et al. designed a series of molecular-scale logic gates by modifying AuNPs with T and C-rich single-stranded DNA. Compared with other logic gates of DNA strand breaks, this strategy of the use of metal ions or complexing agents to control the aggregation and dispersion of AuNPs has good reversibility [128]. Arsenicum (As) and its compounds can induce mutations in organisms and have carcinogenic and teratogenic effects. It is especially important to find a simple and effective way to monitor the As content in the environment. Based on the special interaction between G/T-rich ssDNA and AsO_3_^3−^, Liang proposed a simple, sensitive and selective method using the difference ofmorphological DNA on the AuNPs surface. This method can achieve semi-quantitative analysis, and quantitative analysis by UV-visual spectroscopy with LOD reaching ppb level [129].

The molar absorptivity of AgNPs is 100 times more than that of AuNPs. Therefore, AgNPs can obtain higher sensitivity when used as probes, and have wider applications in the analysis of different targets. Yang’s research group designed a nucleic acid Apt sensor for Hg^2+^ with good selectivity and sensitivity (LOD: 17 nmol L^−1^) [130]. Using chemicals with special functional groups to targetly prepare various AgNPs has improved the detection selectivity and sensitivity and expanded its detection fields. Based on the specific reaction of glutathione and Ni^2+^, glutathione-stabilized AgNPs can detect Ni^2+^ as low as 75 μmol L^−1^ [131]. The protonated dopamine can bind on the surface of AgNPs to reduce the surface charge of the NPs, causing the AgNPs agglomeration, thus achieving colorimetric detection of dopamine (LOD: 40 nmol L^−1^) [132]. Under acidic conditions with a pH of 3.8, the positively charged aminobutyric acid can cause the aggregation of AgNPs due to the electrostatic action, resulting in the change in solution color and plasmon resonance absorption peak, further achieving the quantitative detection of aminobutyric acid [133]. The nanomaterials-based visual biosensors can not only meet the requirement of high sensitivity and have the advantages of simple operation, fast signal response and no need for other equipments. This is especially suitable for on-site rapid detection or areas with relatively poor experimental conditions.

#### 3.1.3. Two-Photon Fluorescence Spectrometry

**Figure 9:**
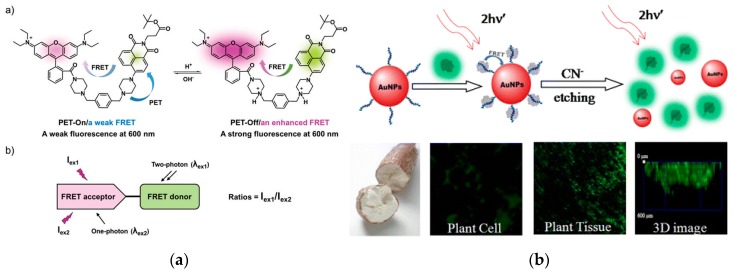
Two-photon fluorescence excitation based on metal NPs. (**a**) High-depth fluorescence imaging using a two-photon FRET system for mitochondrial pH. Reproduced with permission from [139]. Copyright Chemical communications, 2018. (**b**) Nanosensor based on peptide-mediated graphene quantum dots/AuNPs hybrid for two-photon CN^−^ sensing and imaging in plant tissues. Reproduced with permission from [140]. Copyright ACS applied materials & interfaces, 2015.

Compared with single-photon excitation, the two-photon fluorescence excitation technique has low background interference and good penetrability in organisms, and can be more effectively applied to deeper biological tissue research [134,135]. The analysis of the two-photon fluorescence characteristics of noble metal nanomaterials is a new areain recent years. Compared to the traditional organic dye molecules, the two-photon fluorescence technology of noble metal NPs has better chemical stability and light stability [136]. Nano Au and Ag rods have high two-photon fluorescence signals and can be used for imaging and sensing [137,138]. Chang et al. developed a fluorescent pH probe capable of two-photon excitation and far-visible-emission based on fluorescence resonance energy transfer (FRET), composed of naphthalimide-piperazine-rhodamine, which exhibited a pH-dependent reversible and fast ratiometric fluorescence change in the rhodamine emission [139] (Figure 9a). Yang and the co-workers constructed a peptide-mediated graphene quantum dots/AuNPs hybrid nanosensor to for sensing and imaging of CN^−^ in plant tissues. Due to the two-photon properties of graphene quantum dots and the high quenching efficiency of AuNPs, the sensor can achieve a LOD of 0.52 μmol L^−1^ [140] (Figure 9b).

Two-photon fluorescence effect enhanced by the aggregation of noble metal nanomaterials can also be used in sensing detection [141]. Xu et al. have modified AgNPs using a thrombin Apt. When the target thrombin exists, a G-4 complex structure is formed and detached from the surface of AgNPs to cause its aggregation. This strategy has extremely high selectivity and good sensitivity (LOD: 0.1 nmol L^−1^) [142]. Zhao et al. incorporated the Ru(bpy)_3_^2+^ complex into nanoscale metal-organic frameworks (MOFs) to develop a quenching electrochemiluminescence immunosensor for insulin detection. Owing to the steric confinement effect of MOF pores, the quantum yield, luminescence lifetime and two-photon fluorescence intensity were enhanced larger, resulting in the LOD for insulin achieving to 1 ng L^−1^ [143]. Yang et al. have compared the aggregation-enhanced two-photon fluorescence effect of Au@Ag core-shell structures with different shell thicknesses. The results showed that when the thickness of Ag shell was 3.5 nm, the fluorescence enhancement factor was the highest, reaching 840 times, which is very important for improving the detection sensitivity [144]. It is reasonable to believe that two-photon fluorescence detection based on noble metal nanomaterials will increase with the increasing attention, and has the potential in the areas related to human health such as molecular detection, disease diagnosis and treatment.

### 3.2. Electrochemical Sensor

As an important branch of analytical science, electrochemical sensing technology can meet the requirements of miniaturization, integration, online and real-time detection in life analysis, which has important applications in clinical diagnosis, environmental monitoring and food safety [145,146]. Around the preparation of noble metal nanomaterials with different properties, researchers have studied their applications in electrochemical analysis devices [147,148]. Noble metal nanomaterials have large specific surface areas, many surface active sites, strong adsorption capacity and good biocompatibility and stability, and can be used for immobilization and labeling of biologically active molecules, being highly valued in electrochemical analysis [149].

**Figure 10:**
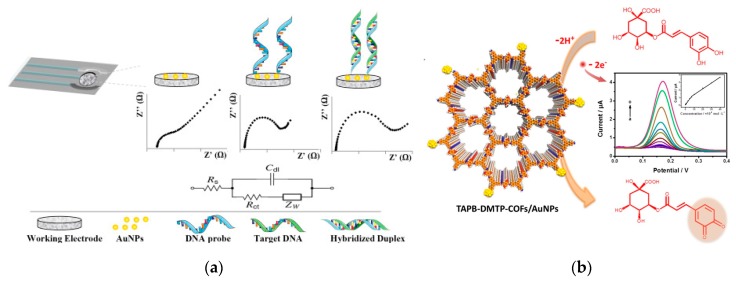
Electrochemical sensor based on AuNPs. (**a**) DNA hybridization sensor based on AuNP- modified SPCE for nucleic acid detection. Reproduced with permission from [153]. Copyright Analytica chimica acta, 2019.; (**b**) The electrochemical probe based on TAPB-DMTP-COFs/AuNPs for chlorogenic acid detection. Reproduced with permission from [154]. Copyright Sensors and Actuators B: Chemical, 2018.

AuNPs have special biocatalytic ability, minimal apparent activation energy, non-toxicity and good biocompatibility, and provide suitable microenvironment for living biological cells, and amplify the analytical signal of electrochemical sensors [150,151]. Xia et al. have developed a simple and convenient cell-based electrochemical biosensor using AuNPs for the evaluation of the individual and combined toxicity of deoxynivalenol, zearalenone, and aflatoxin B_1_ (AFB_1_) on Hep G2 cells [152]. Khater et al. electrodeposited AuNPs on a screen-printed carbon electrode and applied for the electrochemical detection of plant virus, which allows to efficiently immobilize thiolated ssDNA probes as well to enhance the electrode conductivity [153] (Figure 10a). Zhang et al. synthesized novel AuNPs-doped TAPB-DMTP-COFs (TAPB, 1,3,5-*tris*(4-aminophenyl) benzene; DMTP, 2,5-dimethoxyterephaldehyde; COFs, covalent organic frameworks) composite using COFs as the host matrix to support the growth of AuNPs. This novel composite presented a good electrocatalytic activity toward the oxidation of chlorogenic acid, displaying a wide linear range (1.0 × 10^−8^–4.0 × 10^−5^ mol L^−1^), low detection limit (9.5 × 10^−9^ mol L^−1^) as well as a good repeatability (4.1% in 2.0 × 10^−5^ mol L^−1^ concentration after ten scanning rounds) [154] (Figure 10b).

**Figure 11:**
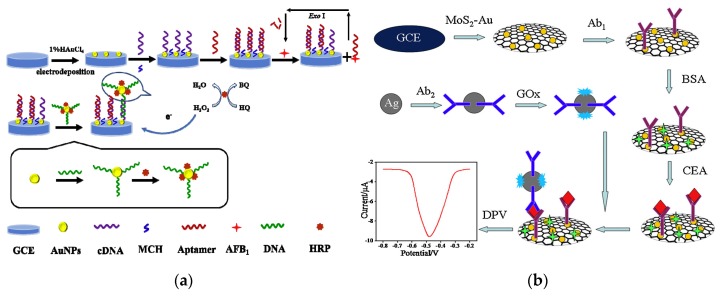
(**a**) Electrochemical aptasensor based on DNA-AuNPs-HRP nanoprobes for detection of AFB_1_. Reproduced with permission from [155]. Copyright Food Control, 2020. (**b**) AgNPs-labeled immunosensor based on MoS_2_-Au composite film. Reproduced with permission from [163]. Copyright Sensors and Actuators B: Chemical, 2015.

Functionalized AuNPs that can significantly enhance and amplify electrochemical signals can be applied to manufacture a variety of electrochemical sensing devices with high sensitivity, good selectivity, high reliability and low cost. Hui et al. reported a electrochemical aptasensor for the detection of AFB_1_ [155] (Figure 11a). The complementary DNA (cDNA) of AFB_1_ Apt was immobilized on the surface of the AuNPs modified electrode. Apt can be bind to cDNA through specific base pairs. When AFB_1_ is present, it can bind to Apt specifically, causing Apt to separate from the electrode surface and produce ssDNA. After adding the DNA-AuNPs-HRP nanoprobes, it will bind to cDNA on the electrode surface and HRP can catalyze redox reactions to produce strong electrochemical signals. Due to the dual amplification effect of AuNPs and HRP, the LOD for AFB_1_ was as low as 0.33 ng L^−1^.

Protein molecules such as enzyme and antibody can bind to the surface of AuNPs through Au–S bond, electrostatic and hydrophobic interactions. The formed complex can maintain the biological activity for a long time, which can significantly improve the sensing stability. Combined with electrochemical technology, AuNPs can analyze and detect a variety of substances. Additionally, AuNPs or AgNPs can combined with other functional materials such as carbon nanotubes [156,157], graphene [158,159] and quantum dots [160], which can enhance the photoelectric response of the sensing interface and improvethe sensitivity. Peng et al. used AuNPs-carbon aerogel and ionic liquid to develop a biocompatible system of hemoglobin. The electrocatalytic property of the constructed biosensor was investigated to have fast responses (within 7 s), good dynamic response ranges and low LOD (2.0 μmol L^−1^ for H_2_O_2_ and 1.3 μmol L^−1^ for NO_2_^−^) [161]. H_2_O_2_ is a commonly used oxidant and disinfectant in modern industry, and has important applications in the field of food and waste water treatment. AgNPs have good electron transfer capability and high electrocatalytic efficiency and can be used in the construction of enzyme-free H_2_O_2_ sensors [162]. Wang et al. reported an AgNPs-labeled immunosensor based on MoS_2_-Au composite film for carcino-embryonic antigen detection [163] (Figure 11b). The MoS_2_-Au composite film has good catalytic activity toward H_2_O_2_. And the AgNPs were used to stabilize the second antibody and glucose oxidase. The whole detection process was performed with a linear range of 1 ng L^−1^–50 μg L^−1^ and the LOD of 0.27 ng L^−1^.

### 3.3. Other Kind of Sensors

SERS is a highly sensitive analytical strategy to study the interaction between molecules and metal surfaces [164,165]. Generally, the surface enhancement effect of metal surface on the Raman signal of target is as high as 10^3^–10^5^ times, while Au and Ag nanomaterials with special structure can obtain higher enhancement factors, which are enough to detect single molecule or DNA chain. Therefore, SERS has become an important tool for the detection of biomacromolecules or small molecules [166,167,168]. Because organic pollutants, DNA, heavy metal ions and other targets cannot be effectively adsorbed on the surface of noble metal nano-substrate, it is difficult to detect by SERS directly. Usually, a substance capable of interacting with a target is modified on the precious metal nano-substrate to achieve the targetfixation and SERS detection.

**Figure 12:**
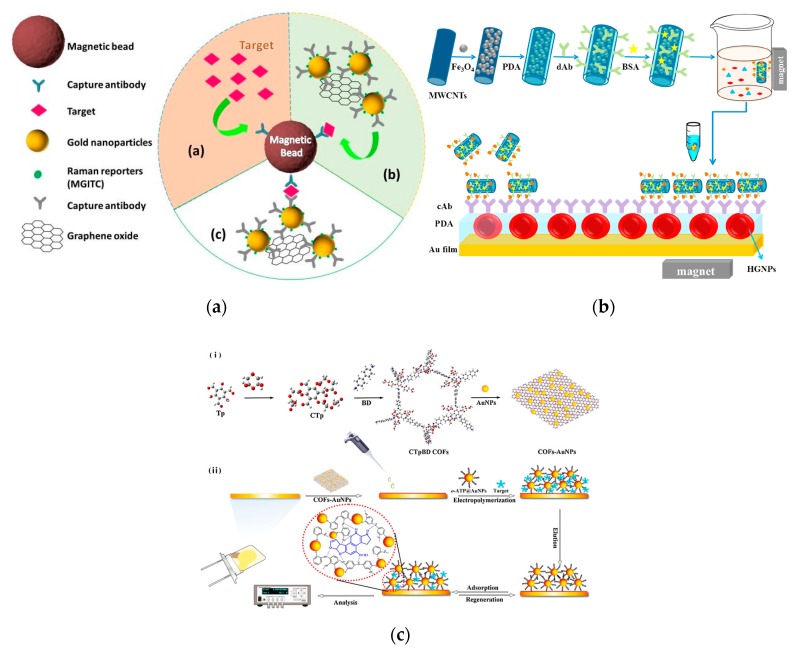
(**a**) The SERS strategy based on GO/AuNPs signal amplification for quantification of cTnI [169]. (**b**) The SPR biosensor based MMWCNTs-PDA immune probe for the detection of cTnI [171]. (**c**) QCM sensor based on COFs-AuNPs for the determination of AFB_1_ [172].

Fu et al. developed a novel signal amplification SERS platform for recognition and determination of cardiac troponin I (cTnI) using AuNPs, graphene oxide (GO) and MBs. Antibody/Raman reporters labeled AuNPs-functionalized GO were applied as both SERS nanotags and signal amplification carriers. Due to the strong SERS enhancement ability and the binding chance with cTnI of GO/AuNPs complexes, the proposed SERS-based immunoassay achieved a high sensitive LOD (5 pg mL^−1^) and a good linearity in a range of 0.01–1000 ng mL^−1^ [169] (Figure 12a). Chen et al. constructed a high-sensitive and low-power theranostic nanosystem that combines with synergistic photothermal therapy and SERS mapping. Due to the “hot spots” effects by the arrangements of AuNPs in the nanochannels of mesoporous silica, it displays high sensitivity to rhodamine 6G solution (LOD: 10^−8^ mol L^−1^). At the same time, a high photothermal therapy efficiency of near-infrared laser was achieved at low power density (0.5 W cm^−2^) because of the synergistic effect from the conjugated AuNPs and rGO nanosheets [170].

The LSPR effect of noble metal nanomaterials is closely related to the size, shape and dielectric constant of surrounding environment. Small ratios or changes in the sharpness of the edges will have an effect on the LSPR effect, causing a change in the absorption or scattering of light. This is the basis for the construction of sensor devices or imaging studies of noble metal nanomaterials [173,174,175]. Wu et al. evaporated thin Au film on a glass slate as SPR sensing film and further modified using hollow AuNPs and polydopamine (PDA). This modified AuNPs/PDA film was used to self-polymerize with dopamine and direct immobilization of capture antibody, significantly enhancing the sensitivity of SPR immunoassay. The LOD for cTnI achieved to 1.25 ng mL^−1^, being 1000-fold lower than that using traditional SPR immunoassay [171] (Figure 12b). This research group also fabricated a similar AuNPs/PDA SPR immunosensor for cardiac biomarker troponin I (cTropI) with LOD of 38 ng mL^−1^, which is 67-fold lower than that of SPR biosensor only using AuNPs [176]. Kabiraz et al. used AuNPs to label the antibody to develop an indirect competitive SPR immunoassay to detect the analyte with a small molecule (clenbuterol) [177]. The SPR immunoassay has an extremely low LOD (0.05 pg mL^−1^), which was 40-fold lower than that using unlabeled antibody. This study systematically evaluated the affinity constants of the surface immunoreaction and the premixed solution, and proposed a method to effectively improve the sensitivity of small molecules detected by SPR. In the construction of piezoelectric sensors, noble metal nanomaterials can also be employed to immobilize biological and biomimetic recognition molecules [178,179]. Kwak and Lee fabricated a piezoelectric immunosensor using AuNPs for signal amplification to sensitive detect prostate-specific antigen (PSA) in human serum. The LOD of PSA immunoassay achieved to 48 pg mL^−1^, which was >14 times lower than that without using AuNPs [180]. Gu et al. developed a quartz crystal microbalance (QCM) sensor based AuNPs doped molecularly imprinted biomimetic layer and COF composite to detect aflatoxin B_1_. It was demonstrated that the COFs-AuNPs substrate and the 3D structure of biomimetic matrix endowed more recognition sites, achieving low LOD of 2.8 pg mL^−1^ [172] (Figure 12c).

## 4. Future Applications

In recent years, various noble metal nanomaterials with different properties were synthesized, and their applications in sensing fields have become increasingly popular, leading to a current research hotspot. According to the current status of relative research, the application of noble metal nanomaterials in the following aspects will be studied in depth.

(1) For fluorescence imaging. The noble metal NCs with strong fluorescence emission, good light resistance and biocompatibility, can overcome the shortcomings of heavy metal toxicity and biodegradation in the current imaging process, which have broad application prospects.

(2) For nanotherapy. Noble metal NPs can safely and efficiently transport drug molecules to target tissues or organs, which not only reduces the adverse reactions of drugs, but also overcomes the natural biological barrier of the organism. The LSPR of some NPs can be regulated to the near-infrared region for photothermal therapy. The use of imaging and laser irradiation technology can realize the integration of diagnosis and treatment of serious diseases.

(3) As a molecular scale. By monitoring the plasma frequency shift caused by the assembly of nano-metal particles, the distance among particles can be changed, realizing the control of some reaction kinetic processes in biological systems and determination of biological macromolecules.

(4) Antibacterial properties. Nano Ag as an antibacterial material is an important research topic. Ag nanomaterials with stable performance and high antibacterial efficiency are prepared and dispersed in products such as plastic to achieve online sterilization and disinfection, having a broad application prospect.
